# A database of annual atmospheric acid and nutrient deposition to China’s forests

**DOI:** 10.1038/sdata.2018.223

**Published:** 2018-10-16

**Authors:** Enzai Du

**Affiliations:** 1 State Key Laboratory of Earth Surface Processes and Resource Ecology, Faculty of Geographical Science, Beijing Normal University, Beijing, 100875, China; 2 School of Natural Resources, Faculty of Geographical Science, Beijing Normal University, Beijing, 100875, China

**Keywords:** Atmospheric chemistry, Element cycles

## Abstract

Anthropogenic emissions have substantially altered atmospheric acid and nutrient deposition in China. Understanding the status and characteristics of acid and nutrient deposition to China’s forests is crucial to assess the consequent impacts, and to better guide forest management options. Based on a comprehensive literature review, here I present an updated database for annual acid and nutrient deposition during the period 1991-2015 in China’s forests (CFAND 2.0). The database includes information from 56 forested sites on the water fluxes of bulk precipitation and throughfall, the concentrations of H^+^ (pH), dissolved inorganic nitrogen (N) (NH_4_^+^ and NO_3_^−^), sulfur (S), dissolved phosphorus (P), potassium (K^+^), calcium (Ca^2+^) and magnesium (Mg^2+^) in bulk precipitation and throughfall, and the fluxes of dissolved inorganic N, S, dissolved P, K^+^, Ca^2+^ and Mg^2+^ in bulk deposition and throughfall. This database will help to understand the spatial patterns of acid and nutrient deposition, validate modelling results of acid and nutrient deposition and assess the ecological effects of acid and nutrient deposition in China’s forests.

## Background & Summary

As a result of rapid industrial, agricultural and urban development, anthropogenic emissions of acid precursors (e.g., NO_x_, NH_3_ and SO_2_) have substantially enhanced acid deposition in China and aroused increasing concerns about their consequent impacts on ecosystem health and function^1–3^. Although national efforts have successfully curbed SO_2_ emissions since the middle 2000s^4^, large areas in China, especially the southern regions, are still subjected to severe acid deposition^5,6^. Acid deposition may increase the leaching of base cationic nutrients (K^+^, Ca^2+^ and Mg^2+^) from soils and cause plants to rely increasingly on base cationic nutrients from atmospheric deposition^1,7,8^. With the reduction of S deposition, atmospheric nitrogen (N) has contributed increasingly to acid deposition in China^9^. High-level N deposition has resulted in an imbalance of atmospheric N and phosphorus (P) inputs to terrestrial ecosystems in China^10^. Moreover, the enhancement of N deposition in China has been evidenced to alter species composition and result in a loss of species diversity in terrestrial ecosystems^2,3^. Forest covers more than 20% of the land area in China and provides fundamental ecosystem services^11^. Therefore, it is important to understand the current status and characteristics of atmospheric acid and nutrient deposition in China’s forests. This is crucial to assess the consequent changes in forest ecosystem services, and to better guide management options.

Current monitoring networks in China provide very limited information on acid and nutrient deposition in China’s forests, because these networks include very limited number of forested sites and/or only measures some components of acid and nutrient deposition. For instance, China Agricultural University is running a nationwide N deposition monitoring network, but the network only includes 7 forested sites over China^12^. Although Chinese Ecosystem Research Network (CERN) measures bulk deposition of acid and base cations, it only includes 14 sites in forest environments^13^. Nevertheless, there are increasing individual measurements of bulk deposition and throughfall fluxes of chemical elements in China’s forests^6,10,14,15^. These measurements allow a more comprehensive assessment of atmospheric acid and nutrients inputs to China’s forests.

**Figure 1:**
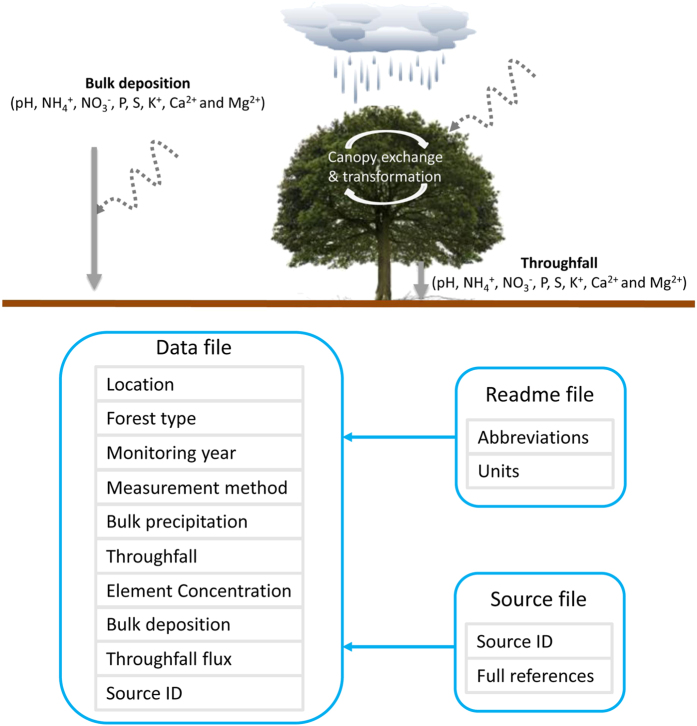
A general view of bulk deposition and throughfall flux in forest ecosystem and the framework of the CFAND 2.0 database. Dashed lines indicate a proportion of dry deposition.

Bulk deposition consists of wet deposition and a proportion of dry deposition, while throughfall is a sum of bulk deposition, canopy captured dry deposition and canopy exchange (e.g., uptake of N or leaching of K^+^) and transformation (e.g., biological nitrification^16^) (Fig. 1). By compiling individual reports on element fluxes in bulk deposition and throughfall, I have constructed a database for annual acid and nutrient deposition in China’s forests (CFAND 1.0). Based on this database, the spatial patterns of acid and nutrient deposition in China’s forests have been assessed. The results indicate high levels of bulk deposition and throughfall fluxes of dissolved inorganic N in China’s forests^14^. A further analysis suggests an imbalance of atmospheric N and P inputs to China’s forests, which may lead to a shift towards P limitation^10^. Moreover, relatively high levels of bulk deposition of base cationic nutrients were found in China’s forests, which neutralized a significant proportion of the potential acid load due to N and S deposition^15^. Overall, both bulk deposition and throughfall fluxes of N, S, P, K^+^, Ca^2+^ and Mg^2+^ have been evidenced to show a significant increase with closer distance to the nearest large cities, suggesting a substantial anthropogenic alteration of the regional cycling of these elements^6,10,14,15^.

The World Meteorological Organization (WMO) Global Atmosphere Watch programme (GAW) has recently completed an assessment of global precipitation chemistry and deposition, while this assessment only included data from very limited number of sites in China^17^. The absence of an open-access database of acid and nutrient deposition in China’s forests motivates the update and publication of the CFAND 2.0 database. The CFAND 2.0 database is structured on three files, including a ‘data file’, a ‘readme file’ and a ‘source file’ (Fig. 1). It includes detailed information on the water fluxes of bulk precipitation and throughfall, the concentrations of H^+^ (pH), dissolved inorganic N (NH_4_^+^ and NO_3_^−^), S, dissolved P, K^+^, Ca^2+^ and Mg^2+^ in bulk precipitation and throughfall, and the fluxes of dissolved inorganic N, S, dissolved P, K^+^, Ca^2+^ and Mg^2+^ in bulk deposition and throughfall as well as geographical information (longitude and latitude), forest type, and methods of field sampling and laboratory analysis. This database can be used to analyse the characteristics of annual acid and nutrient deposition, validate modelling results of acid and nutrient deposition and assess the ecological impacts of acid and nutrient deposition in China’s forests. Due to a lack of long-term monitoring data for each site, this database is not able to track changes in acid and nutrient deposition over time.

## Methods

### Database structure

The CFAND 2.0 database is composed of three files (Fig. 1). The ‘data file’ includes the main data values for the water fluxes of bulk precipitation and throughfall, the concentrations of H^+^ (pH), dissolved inorganic N, S, dissolved P, K^+^, Ca^2+^ and Mg^2+^ in bulk precipitation and throughfall, and the fluxes of dissolved inorganic N, S, dissolved P, K^+^, Ca^2+^ and Mg^2+^ in bulk deposition and throughfall as well as geographical information (longitude and latitude), forest type, measurement method, and monitoring year associated with each record (Table 1; Fig. 1). The ‘readme file’ explains the abbreviations used by ‘data file’ and includes information on the units for bulk precipitation, throughfall, element concentrations and fluxes (Fig. 1). The ‘source file’ includes the full reference for each literature source used in the database (Fig. 1).

### Data compilation

By conducting a comprehensive survey of the online library of China National Knowledge Infrastructure (http://www.cnki.net/) and ISI Web of Science (http://isiknowledge.com), data were collected from published literature on the concentrations of H^+^ (pH), dissolved inorganic N (NH_4_^+^ and NO_3_^−^), S, dissolved P, K^+^, Ca^2+^, Mg^2+^ in bulk precipitation and throughfall in China’s forests, as well as information on the water fluxes of bulk precipitation and throughfall, geographical information (latitude and longitude), forest type, sampling frequency, and laboratory analysis method. Specifically, field sampling and laboratory analysis were conducted at different frequencies for different studies (e.g., every precipitation event, weekly or monthly). The concentrations of dissolved inorganic N were measured mainly by a flow injection analyzer or ion chromatography method. The concentrations of S were measured by barium sulfate turbidity method, ion chromatography method or a flow injection analyzer. The concentrations of dissolved P were measured by phosphoantimonylmolybdenum blue spectrophotometric method, or a flow injection analyzer. The concentrations of base cations were mainly measured by an atomic absorption spectrometer or an inductive coupled plasma emission spectrometer.

Monitoring data were included only when the element concentrations in bulk precipitation and throughfall were measured simultaneously. Data were either taken directly from tables in the literature or digitized from figures using a GetData Graph Digitizer (Version 2.25, http://www.getdata-graph-digitizer.com). The values of acid and nutrient concentrations in CFAND 2.0 database were annually averaged. If concentration data at one site were measured for more than one forest stand or available for more than one year in a monitoring study, a volume-weighted mean was calculated and used for further analysis.

Bulk deposition (BPDep, kg ha^−1^ yr^−1^) of each element was calculated by multiplying the volume-weighted mean concentration (BPCon, mg L^−1^) in bulk precipitation with annual bulk precipitation (BP, mm) according to Eq. (1),

(1)BPDep=0⋅01×BPCon×BP

Throughfall flux (TFFlux, kg ha^−1^ yr^−1^) of each element was calculated by multiplying the volume-weighted mean concentration (TFCon, mg L^−1^) in throughfall with annual throughfall (TF, mm) according to Eq. (2),

(2)TFFlux=0⋅01×TFCon×TF

### Code availability

No custom computer code was used to generate the data described in the manuscript.

## Data Records

The data compiled are available in a single dataset (Data Citation 1), which consists of three Microsoft Excel files: the ‘data file’ (CFAND 2.0 Data File) is the main file, the ‘readme file’ (CFAND 2.0 Read Me) explains the abbreviations and units, and the ‘source file’ (CFAND 2.0 Source File) includes the full references used in the database (Fig. 1). The CFAND 2.0 database includes a wealth of monitoring data from Chinese literature (see more details in source file) and at current stage presents the most comprehensive and newly updated data on acid and nutrient deposition in China’s forests.

By compiling reported data from 65 sources (see CFAND 2.0 Source File for more information), CFAND 2.0 database includes information on the bulk deposition and throughfall fluxes of acid and/or nutrients from 56 forested sites across main forest biomes in China (see CFAND 2.0 Data File for more information). All recorded data were measured during the period 1991-2015, although measurements for most sites were conducted for 1-3 years. Specifically, CFAND 2.0 database includes 45 records for pH from 39 sites, 43 records for dissolved inorganic N (NH_4_^+^ and NO_3_^−^) from 37 sites, 40 records for S from 35 sites, 41 records for dissolved P from 26 sites, 71 records for K^+^ from 56 sites, 72 records for Ca^2+^ from 56 sites and 72 records for Mg^2+^ from 56 sites (Table 1). Generally, the geometric mean bulk deposition was much lower than annual throughfall flux for NH_4_^+^-N (8.9 vs. 10.4 kg ha^−1^ yr^−1^), NO_3_^−^-N (5.4 vs. 6.2 kg ha^−1^ yr^−1^), S (25.7 vs. 37.7 kg ha^−1^ yr^−1^), dissolved P (0.49 vs. 0.90 kg ha^−1^ yr^−1^), K^+^ (8.9 vs. 33.6 kg ha^−1^ yr^−1^), Ca^2+^ (25.7 vs. 37.1 kg ha^−1^ yr^−1^) and Mg^2+^ (2.6 vs. 6.7 kg ha^−1^ yr^−1^), respectively. This difference indicates a substantial amount of canopy captured dry deposition. In addition, canopy leaching of base cations might also contribute to the remarkable enrichment in throughfall versus bulk deposition^20,21^. However, throughfall flux of dissolved inorganic N was sometimes lower than bulk deposition, implying an uptake of N by forest canopy^18,19^.

## Technical Validation

All records included in CFAND 2.0 database are based on published material in peer-reviewed journals (80%), books (2%), or theses (18%), and thus most data should be reliable. Each data record has been checked for possible errors and the extreme values being orders of magnitude than the mean value of all sites were excluded. Uncertainties also remain due to the laboratory analysis of element concentrations by different methods. The CFAND 2.0 database has thus included detailed information on the sampling and laboratory analysis methods from original references, which can help users to evaluate the validity and accuracy of data. Nevertheless, the early version of this database (CFAND 1.0) has been reviewed by international peers and several papers associated with this database have been published^6,14,15^. Overall, CFAND 2.0 database provides high-quality open-access information on annual acid and nutrient deposition in China’s forests.

## Additional information

**How to cite this article:** Du E. A database of annual atmospheric acid and nutrient deposition to China’s forests. *Sci. Data*. 5:180223 doi: 10.1038/sdata.2018.223 (2018).

**Publisher’s note:** Springer Nature remains neutral with regard to jurisdictional claims in published maps and institutional affiliations.

## Supplementary Material



## Figures and Tables

**Table 1:** A summary of the input data on atmospheric acid and nutrient deposition in China’s forests.

Item	Components	No. of sites	No. of records
Bulk deposition	pH	39	45
	DIN	37	43
	NH_4_^+^	37	43
	NO_3_^-^	37	43
	S	35	40
	P	26	41
	K^+^	56	71
	Ca^2+^	56	72
	Mg^2+^	56	72
Throughfall	pH	39	45
	DIN	37	43
	NH_4_^+^	37	43
	NO_3_^-^	37	43
	S	35	40
	P	26	41
	K^+^	56	71
	Ca^2+^	56	72
	Mg^2+^	56	72
